# *Schistosoma haematobium* infection and morbidity risk factors for pre-school age children in western Angola: A knowledge, attitudes and practices survey

**DOI:** 10.1371/journal.pntd.0011650

**Published:** 2023-10-16

**Authors:** Raquel Sánchez-Marqués, Fernando Salvador, Cristina Bocanegra, Arlette Nindia, Zeferino Pintar, Joan Martínez, Sandra Aixut, Agostinho Pessela, Sheila Ramírez-Arroyo, Aina Farrés, María Chopo, Silvia Izquierdo, Santiago Mas-Coma, María Dolores Bargues, Israel Molina

**Affiliations:** 1 Departamento de Parasitología, Facultad de Farmacia, Universidad de Valencia, Valencia, Spain; 2 Centro de Investigación Biomédica en Red de Enfermedades Infecciosas (CIBERINFEC), Instituto de Salud Carlos III, Madrid, Spain; 3 Tropical Medicine Unit Vall d’Hebron-Drassanes, Infectious Diseases Department, Vall d’Hebron University Hospital, PROSICS Barcelona, Barcelona, Spain; 4 Hospital Nossa Senhora da Paz, Cubal, Angola; Colorado State University, UNITED STATES

## Abstract

**Background:**

Urogenital schistosomiasis is one of the most prevalent parasitic diseases in sub-Saharan Africa. It is a poverty-related disease conditioned by behavioural practices.

**Methods:**

Our objective is to evaluate the awareness, mindset and habits about urogenital schistosomiasis in the community of Cubal (Angola), as well as its association with infection and urinary tract morbidity in pre-school age children. A cross-sectional study of knowledge, attitudes and practices at home was conducted between February and May 2022 with 250 participants.

**Results:**

Overall, 93.6% of those surveyed had some prior knowledge about schistosomiasis and, among all the symptoms associated with this disease, blood in the urine was the best known (54.4%). Nevertheless, 57.6% obtained a medium knowledge score. Regarding attitude, the majority of respondents had a high attitude score (79.2%) with 96.0% willing to participate in mass drug administration campaigns. Laundry in the river was the most common risk practice (61.2%) and 55.2% out of the total were classified with a low practice score.

**Conclusion:**

Low knowledge about symptoms and transmission by caregivers was the outstanding risk factor for infection in pre-school age children (OR = 16.93, 95%CI: 3.93–72.82), and lack of knowledge that avoiding entering the river prevents schistosomiasis was the main risk factor for morbidity in PSAC (OR = 8.14, 95%CI: 1.14–58.25).

## Author summary

Schistosomiasis is one of the most prevalent parasitic diseases and, due to its transmission by infested water, it is particularly linked to the poverty of the populations suffering from it, since most of them do not have easy access to running water. The main activities aimed at interrupting the parasite cycle require community participation and acceptance for their implementation, and for this purpose, being aware of the practical, cultural and social determinants of the disease in a particular population is essential. In this sense, KAP (knowledge, attitudes and practices) surveys may reveal misconceptions or misunderstandings that could represent obstacles to the health empowerment of the population at risk and potential barriers to behaviour changes. This study shows the results of a baseline KAP survey in a region in Angola of high endemicity for urinary schistosomiasis, showing that the population’s knowledge of the conditioning factors of the disease is intermediate and that poor knowledge and risk practices of caregivers of preschool children can represent a risk factor both for the infection of the children and for the development of the disease and its associated morbidity.

## Introduction

Schistosomiasis is a poverty-related disease with 250 million people infected, of which 201.4 million are in sub-Saharan Africa and 200,000 people dies annually [1]. Global Burden of the Disease (2019) determined that schistosomiasis is the cause of 3.3 Disability-Adjusted Life Years (DALY’s), which can result in loss of family income due to the inability of infected family members to work, learning difficulties in children or social exclusion of women. Its control and eradication relays on four main actions that seek to interrupt the life cycle of the parasite: mass drug administration (MDA) with 40 mg/kg of praziquantel, snail vector control, water and sanitation and hygiene (WASH), and health promotion. MDA is recommended for children older than two years and pregnant women in endemic populations [2,3], with the objective of reaching at least 75%. MDA interrupts the accumulative morbidity caused by the disease, which can lead to serious damage to the urinary tract and even death if left untreated. Nevertheless, reinfection tends to occur within a year of treatment, especially in school-age children (SAC).

Human schistosomiasis is caused by trematodes of the genus *Schistosoma*, of which six species are capable of causing the disease in humans [4]: *S*. *mansoni*, *S*. *japonicum*, *S*. *mekongi*, *S*. *guineensis*, *S*. *intercalatum*, and *S*. *haematobium*. Given their life cycle, trematodiases are closely linked to social and behavioural practices [5,6]. Since the vector of schistosomiasis is a freshwater snail, people become infected by coming into contact with infested waterbodies. Therefore, the patterns of contact with water define the risk of infection of populations, highlighting the endangered groups of SAC together with certain professions such as farmers, fishermen or housewives [4,7,8]. Recently, the World Health Organization (WHO) has included pre-school age children (PSAC) in the high-risk groups recommended for MDA [3], since many studies describe a high prevalence of urogenital schistosomiasis in this group of age [9,10]. They usually become infected while their caregivers use the river water for daily tasks [11]. Because of the social nature of its transmission, health empowerment of population at risk through health promotion, in combination with other strategies, can lead to behaviour change that reduces the overall prevalence and the progression of the infection towards an acute mode. Its success is based on a good understanding of the practical, cultural and social determinants of the specific area where the intervention will take place. For this purpose, surveys on aspects such as knowledge, attitudes and practices (KAP) are usually carried out in the intervention areas.

Angola is an African country with a national poverty rate of 41%, reaching more than 90% for almost half of Angolan municipalities [12]. Its official prevalence of urogenital schistosomiasis is 28%, while intestinal schistosomiasis remains residual throughout the country [13]. Benguela is one of the 18 Angolan provinces and is ranked as the second most prevalent province in the country [14]. In Cubal, a municipality of Benguela, a 61% urogenital schistosomiasis prevalence among SAC was previously described [15].

Therefore, our objective is to provide the baseline on the KAP regarding urinary schistosomiasis in Cubal, as well as to determine risk factors associated with the infection and morbidity of PSAC in relation to the knowledge and practices of their caregivers.

## Methodology

### Ethics statement

This study was approved by the ethics committee of the Ministério de Saúde de Angola (MINSA) (n°41/2021) and was conducted in compliance with the principles of the Declaration of Helsinki, Good Clinical Practice guidelines, and local regulatory requirements. Each participant was informed about the type of study and the purpose of its outcomes. The participants (or caregivers in the case of children) signed an informed consent for the inclusion of their children in the study. Caregivers were considered to be parents or guardian of the children. The informed consent and the survey were provided in both Portuguese and the local language (Umbundu).

### Study design

This study was a quantitative, cross-sectional household survey that assessed KAP towards urogenital schistosomiasis and their caregivers’ behavioural risk factors for PSAC infection and morbidity. It took place between February and May 2022 in Cubal (Angola).

### Study site and sample size

The province of Benguela is made up of 10 municipalities, of which six have a poverty rate above 80%. One of them is the rural municipality of Cubal with an estimated population of 322,000 inhabitants and a poverty rate of 87.3%. It has a lake and a river that surrounds the entire southern part of the municipality used for fishing, bathing, and laundry. Participants were recruited in the course of a parallel study with the aim of evaluating the prevalence and morbidity associated with urogenital schistosomiasis in children under 5 years of age through urine filtration and ultrasound examination. This study was carried out in the facilities of Hospital Nossa Senhora da Paz in Cubal. As the target population was PSAC, traditional school recruitment was not possible and therefore a call was made in churches and markets to report on the ongoing study. On the other hand, children accompanying relatives to the hospital were also recruited. All children under 5 years of age whose parents voluntarily signed the informed consent were included in that study, resulting in a prevalence of urogenital schistosomiasis of 30.2% and a urinary tract (UT) morbidity of 54.5% among infected PSAC. All caregivers of the children included in the PSAC study were offered to participate in the surveys and were recruited (when more than one caregiver was present with the children, only the one who spent the most time caring for the child was surveyed). In addition, active recruitment was carried out in all the neighbourhoods of Cubal, arbitrarily selecting people on the streets who voluntarily wanted to answer the surveys.

### Survey questionnaire

The questionnaire was designed in order to quantitatively assess the KAP towards schistosomiasis as well as the socioeconomic situation of the household. Since there is no model for such a KAP survey on schistosomiasis, we designed it based on the WHO guidelines for KAP surveys [16]. To assess the socioeconomic situation of household, the Multiple Indicator Cluster Survey (MICS3) [17] was included, and the overcrowding index was defined as houses where there are more than three people per habitable room [18]. The scoring and interpretation of the survey are shown in Table 1. It was divided into four blocks: 1) demographic data, 2) knowledge, which included three subblocks, transmission and symptoms (here named jointly as disease knowledge), prevention and treatment, 3) attitudes, and 4) household socioeconomic situation. The practice block had a nominal scale design ranging from “never” to “every day”. The attitude block included a Likert-scale question (from 0 to 5) about trust in doctors. All other items were asked as an open question in order to ensure that participants expressed their real thoughts and were not influenced by predefined answers.

**Table 1:** Scoring and ranking of survey answers.

	Knowledge				Attitude	Practice	Socioeconomic
	Disease	Prevention	Treatment	Schistosomiasis knowledge			
**Scoring**	Correct answer = 1 Incorrect answer = 0	Correct answer = 1 Incorrect answer = 0	Correct answer = 1 Incorrect answer = 0	Disease score + Prevention score + Treatment score	Yes = 1 No = 0 Likert ≤ 3 = 0 Liker > 3 = 1	Yes = 0 No = 1	UN, 2005
**Range**	0–14	0–5	0–3	0–22	0–6	0–10	0–26
**Classif**.	0–5: low 6–10: average 11–14: high	0–1: low 2–3: average 4–5: high	0–1: low 2: average 3: high	0–6: low 7–14: average 15–22: high	0–2: low 3–4: average 5–6: high	0–3: low 4–6: average 7–10: high	0–8: low 9–17: average 18–26: high

### Data analysis

The total scores were divided into thirds to classify between low, medium and high (Table 1). The results of the survey were described using descriptive statistics, while Pearson’s Chi-squared test was used to analyse the correlation between variables. Odds Ratio (OR) with a 95% confidence interval was used to assess the risk factors for PSAC. The prevalence and morbidity results of the previous PSAC study were integrated to assess the risk factors of their caregivers. All statistical analyses were performed using SSPS.

## Results

### Study sample

Table 2 shows the demographic data of the survey participants. 250 people were involved in the study, aged between 15 and 61 years with a mean age of 31.2 (standard deviation, SD = 10.08) and the majority were women (62.4%). Most of the participants were married (61.2%) and slightly more than half knew how to write and read in Portuguese (59.6%). Regarding the education level, 23.6% never went to school while only 3.2% have studied at the university. The most common nearby water body was the river, as 33.6% stated that they lived less than 15 minutes from the river, while only 9.2% lived at the same distance from the lake. Of the total, 183 (73.2%) were caregivers who agreed to include their children under 5 years of age in the aforementioned parallel study on the prevalence and morbidity of urogenital schistosomiasis.

**Table 2:** Demographic characteristics of the participants.

	Number of participants (N = 250) N (%)
**Gender**	
Female	156 (62.4)
Male	94 (37.6)
**Age**	
<40	206 (82.4)
≥40	44 (17.6)
**Marital status**	
Married	153 (61.2)
Single	83 (33.2)
Widow	14 (5.6)
**Literacy level**	
Can write and read	149 (59.6)
Cannot write and read	101 (40.4)
**Higher educational level**	
None	59 (23.6)
Primary	64 (25.6)
Secondary	69 (27.6)
College preparatory	18 (7.2)
Professional training	32 (12.8)
University	8 (3.2)
**Distance to the river**	
<5 min	28 (11.2)
5–15 min	56 (22.4)
16–30 min	106 (42.4)
≤30 min	60 (24.0)
**Distance to the lake**	
<5 min	4 (1.6)
5–15 min	19 (7.6)
16–30 min	13 (5.2)
≤30 min	214 (85.6)
**Caregivers**	183 (73.2)

### Urogenital schistosomiasis knowledge

Table 3 summarizes the urogenital schistosomiasis knowledge of the population. 234 people (93.6%) declared knowing that schistosomiasis is a disease. The majority indicated the street (47.6%) and the hospital (38.0%) as their main source of information about it. Blood in the urine was the most well-known symptom (54.4%), followed by abdominal pain (30.4%), and 58.0% of them believed that it is a dangerous disease that can cause death. Water activities in rivers were identified as a source of infection by 32.8% of the participants and only 2.8% a freshwater snail as a vector of the disease. On the other hand, there is a popular belief among the community that people become infected when eating sugar cane, which is represented in the survey with 20.8% of responses. Regarding prevention, 72.4% stated that schistosomiasis infection can be avoided. The most popular prevention measure among those surveyed was taking prophylactic medication (35.6%), followed by refraining from entering the river (20.4%). The greatest knowledge about schistosomiasis among the respondents referred to the treatment. Almost all of them (91.8%) assured that they can be cured either with modern medicine tablets (80.0%) or traditional medicine (16.8%). The overall mean knowledge score was 7.09 (SD = 3.25), with the majority (57.6%) having a medium knowledge score and 42% a low knowledge score.

**Table 3:** Knowledge about schistosomiasis, prevention and treatment.

Variable	Female (N = 156) N (%)	Male (N = 94) N (%)	Total (N = 250) N (%)
**DISEASE**			
**Heard about**	144 (92.3)	90 (95.7)	234 (93.6)
**Source of information**			
School	16 (10.3)	17 (18.1)	33 (13.2)
Hospital	59 (37.8)	36 (38.3)	95 (38.0)
Street	76 (48.7)	43 (45.7)	119 (47.6)
Church	12 (7.7)	20 (21.3)	32 (12.8)
Home	22 (14.1)	27 (28.7)	49 (19.6)
Media	-	2 (2.1)	2 (0.8)
**Symptoms**			
Abdominal pain	50 (32.1)	26 (27.7)	76 (30.4)
Blood in urine	66 (42.3)	70 (74.5)	136 (54.4)
Fever	11 (7.1)	9 (9.6)	20 (8.0)
General discomfort	3 (1.9)	2 (2.1)	5 (2.0)
**Cause of death**	84 (53.8)	61 (64.8)	145 (58.0)
**How to get infected**			
River bath	44 (28.2)	38 (40.4)	82 (32.8)
Laundry	5 (3.2)	2 (2.1)	7 (2.8)
Drink water	9 (5.8)	10 (10.6)	19 (7.6)
Fishing	1 (0.6)	2 (2.1)	3 (1.2)
Eating sugar cane	25 (16.0)	27 (28.7)	52 (20.8)
Snail as the vector	2 (1.3)	5 (5.3)	7 (2.8)
**Who can get infected**			
Everyone	107 (68.6)	72 (76.6)	179 (71.6)
Only boys	3 (1.9)	2 (2.1)	5 (2.0)
Only children	11 (7.1)	10 (10.6)	21 (8.4)
**It can be prevented**	104 (66.6)	77 (81.9)	181 (72.4)
**How to prevent it**			
Avoid the river	25 (16.0)	26 (27.7)	51 (20.4)
To boil water	15 (9.6)	8 (8.5)	23 (9.2)
Medication	51 (32.7)	38 (40.4)	89 (35.6)
Not to eat sugar cane	11 (7.1)	18 (19.1)	29 (11.6)
**TREATMENT**			
**It can be cured**	139 (89.1)	89 (94.7)	228 (91.2)
**How can it be cured**			
Traditional medicine	23 (14.7)	19 (20.2)	42 (16.8)
Modern medicine pills	123 (78.8)	77 (81.9)	200 (80.0)
**KNOWLEDGE SCORE**			
Low knowledge score	77 (49.4)	28 (29.8)	105 (42.0)
Average knowledge score	78 (50.0)	66 (70.2)	144 (57.6)
High knowledge score	1 (0.6)	0 (0.0)	1 (0.4)
**MEAN SCORE**	6.6 (SD = 3.2)	7.9 (SD = 3.2)	7.1 (SD = 3.2)

### Attitudes towards schistosomiasis

Most of the participants (87.2%) believe that blood in the urine and abdominal pain is a compelling reason to seek medical care (Table 4). Nonetheless, 58.8% declared that they go to the hospital whenever they feel sick. A considerable number of respondents stated that the hospital is not their first choice because it is too expensive (26.4%) or too far from their residence (5.2%). However, 96% expressed their willingness to participate in an MDA campaign against schistosomiasis. The mean attitude score among the participants was 5.17 (SD = 0.93) and 79.2% obtained a high attitude score.

**Table 4:** Attitude towards schistosomiasis.

Variable	Female (N = 156) N (%)	Male (N = 94) N (%)	Total (N = 250) N (%)
Willing to participate in MDA	150 (96.2)	90 (95.7)	240 (96.0)
Trust in doctors	122 (78.2)	79 (84.0)	201 (80.4)
Worried if found blood in urine	138 (88.5)	80 (85.1)	218 (87.2)
Worried if abdominal pain	136 (87.2)	82 (87.2)	218 (87.2)
**Hospital as first choice**	93 (59.6)	54 (57.4)	147 (58.8)
Why not?			
Hospital is too far	4 (2.6)	9 (9.6)	13 (5.2)
Hospital is too expensive	23 (14.7)	43 (45.7)	66 (26.4)
Prefers traditional medicine	6 (3.8)	13 (13.8)	19 (7.6)
**ATTITUDE SCORE**			
Low attitude score	1 (0.6)	2 (2.1)	3 (1.2)
Average attitude score	32 (20.5)	17 (18.1)	49 (19.6)
High attitude score	123 (78.8)	75 (79.8)	198 (79.2)
**MEAN SCORE**	5.1 (SD = 0.8)	5.2 (SD = 1.0)	5.1 (SD = 0.93)

### Practices related to schistosomiasis

Laundry at the river was the most popular practice during last year with 61.2% of the participants doing it (Table 5), and 32.0% affirmed to do it only during the dry season due to the lack of another source of water. The same situation occurs with having bath in the river (58.8%), while 30.8% do so only in the dry season. Fishing is not a common practice among the community. Therefore, only 15.6% have been in contact with the river because of this practice during the last year, being significantly higher among men (X^2^ = 15.7, *p*-value <0.01). Regarding fetching water from the river to irrigate crops, only 8% of those surveyed do so frequently, but 21.6% need it to continue with their gardening work during the dry season. Only 56 people (19.2%) stated that they carried out a prevention activity against schistosomiasis. Nonetheless, it is worth mentioning that 17 of them said not eating sugar cane is the prevention activity they perform. The mean practice score was 3.5 (SD = 2.24) with 55.2% having a low practice score.

**Table 5:** Practices related to schistosomiasis.

Variable	Female (N = 156) N (%)	Male (N = 94) N (%)	Total (N = 250) N (%)
**To urinate into the river**	75 (48.1)	50 (53.2)	125 (50.0)
**To perform schistosomiasis prevention**	17 (10.9)	31 (32.9)	48 (19.2)
**To have bath at the river**	90 (57.7)	57 (60.6)	147 (58.8)
Only during dry season	50 (32.0)	27 (28.7)	77 (30.8)
Frequently	40 (25.6)	30 (31.9)	70 (28.0)
**To laundry at the river**	103 (66.0)	50 (53.2)	153 (61.2)
Only during dry season	57 (36.5)	23 (24.5)	80 (32.0)
Frequently	46 (29.5)	27 (28.7)	73 (29.2)
**To fetch river water for daily tasks**	80 (51.3)	49 (52.1)	129 (51.6)
Only during dry season	42 (26.9)	23 (24.5)	65 (26.0)
Frequently	38 (24.3)	26 (27.6)	64 (25.6)
**To fetch river water for irrigation**	40 (25.6)	34 (36.2)	74 (29.6)
Only during dry season	25 (16.0)	29 (30.8)	54 (21.6)
Frequently	15 (9.6)	5 (5.3)	20 (8.0)
**To fish**	16 (10.3)	23 (24.5)	39 (15.6)
**PRACTICE SCORE**			
Low practice score	86 (55.1)	52 (55.3)	138 (55.2)
Average practice score	59 (37.8)	27 (28.7)	86 (34.4)
High practice score	11 (7.1)	15 (16.0)	26 (10.4)
**MEAN SCORE**	3.5 (SD = 2.1)	3.4 (SD = 2.4)	3.5 (SD = 2.2)

### Household socioeconomic status

Most of the participants (53.2%) have a medium socioeconomic status, while 31.6% live in a low socioeconomic situation and 174 (69.6%) households were overcrowded (Table 6). Most of those surveyed have to walk less than 15 minutes to fetch water (71.2%) and only 44 people (17.6%) have a piped water supply at their residence. The most popular environment to live in was urban areas (66.8%) and most of the construction materials are sheet metal for the roof, mud for the walls and cement for the floor (69.6%; 47.2%; 57.6%, respectively).

**Table 6:** Socioeconomic characteristic of participants.

Variable	Female (N = 156) N (%)	Male (N = 94) N (%)	Total (N = 250) N (%)
**Number of people contributing to household income**			
None	23 (14.7)	7 (7.4)	30 (12.0)
One	94 (60.3)	69 (73.4)	163 (65.2)
Two	32 (20.5)	2 (2.1)	32 (12.8)
Three or more	7 (4.5)	4 (4.3)	11 (4.4)
**House items**			
Overcrowding	108 (69.2)	66 (70.2)	174 (69.6)
Electricity	52 (33.3)	36 (38.3)	88 (35.2)
Tap water	25 (16.0)	19 (20.2)	44 (17.6)
Radio	51 (32.7)	64 (68.1)	115 (46.0)
TV	42 (26.9)	30 (31.9)	72 (28.8)
**House environment**			
Swampy area	22 (14.1)	8 (8.5)	30 (12.0)
Rural	18 (11.5)	18 (19.1)	36 (14.4)
Urban area	108 (69.2)	59 (62.8)	167 (66.8)
Paved urban area	8 (5.1)	9 (9.6)	17 (6.8)
**Roof**			
Sticks	20 (12.8)	14 (14.9)	34 (13.6)
Sheet metal	114 (73.1)	60 (63.8)	174 (69.6)
Cement	22 (14.1)	20 (21.3)	42 (16.8)
**Walls**			
Sticks	5 (3.2)	7 (7.4)	12 (4.8)
Mud	87 (55.8)	31 (33.0)	118 (47.2)
Blocks	27 (17.3)	29 (30.9)	56 (22.4)
Cement	37 (23.7)	27 (28.7)	64 (25.6)
**Floor**			
Ground	57 (36.5)	32 (34.0)	89 (35.6)
Cement	89 (57.1)	55 (58.5)	144 (57.6)
Tiles	10 (6.4)	7 (7.4)	17 (6.8)
**Cooking material**			
Firewood	33 (21.2)	23 (24.5)	56 (22.4)
Charcoal	42 (26.9)	16 (17.0)	58 (23.2)
Gas	81 (51.9)	54 (57.4)	135 (54.0)
Electricity	0 (0.0)	1 (1.1)	1 (0.4)
**WASH**			
No place to urine	29 (18.6)	26 (27.6)	55 (22.0)
Latrine	75 (48.1)	47 (50.0)	122 (48.8)
Toilet	52 (33.3)	21 (22.3)	73 (29.2)
**Walking time to closest tap water**			
0 min	25 (16.0)	19 (20.2)	44 (17.6)
1–5 min	82 (52.5)	41 (43.6)	123 (49.2)
6–15 min	31 (19.8)	24 (25.5)	55 (22.0)
16–30 min	11 (7.1)	9 (9.6)	20 (8.0)
> 30 min	7 (4.5)	1 (1.1)	8 (3.2)
**SOCIOECONOMIC SCORE**			
Low score	51 (32.7)	28 (29.8)	79 (31.6)
Average score	82 (52.6)	51 (54.3)	133 (53.2)
High score	23 (14.7)	15 (16.0)	38 (15.2)
**MEAN SCORE**	11.3 (SD = 5.6)	11.6 (SD = 6.5)	11.4 (SD = 5.9)

### Risk factors for PSAC infection and morbidity regarding their caregivers

**Fig 1:**
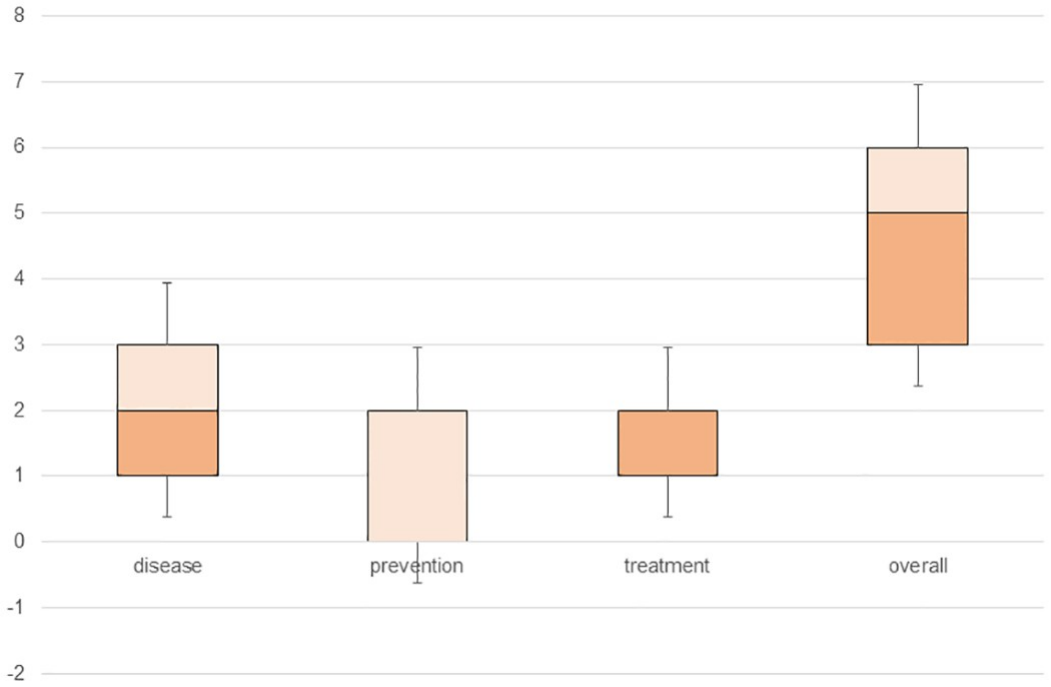
Schistosomiasis knowledge score statistics among caregivers of infected PSAC.

**Fig 2:**
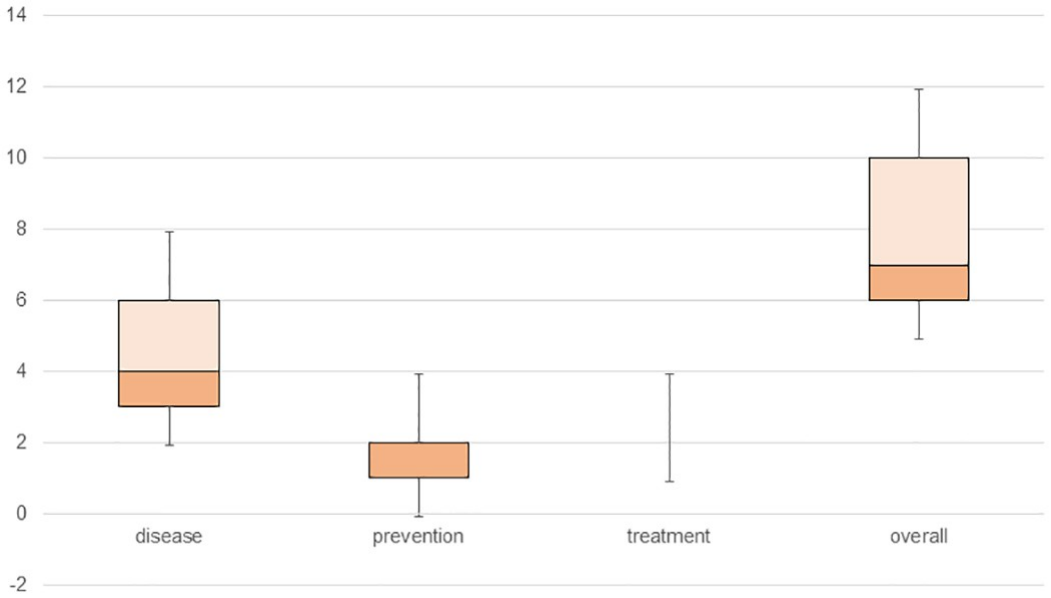
Schistosomiasis knowledge score statistics among caregivers of non-infected PSAC.

As shown in Table 7, a poor knowledge about schistosomiasis was a risk factor for schistosomiasis infection in PSAC (OR = 7.45, 95%CI: 3.72–14.91). More in detail, poor knowledge about how it is transmitted and its symptoms represents the highest risk factor (OR = 16.93, 95%CI: 3.93–72.82). In fact, as can be seen in the statistics (maximum and minimum scores, lower quartile, upper quartile, and median) plotted in Figs 1 and 2, both the overall knowledge score and that corresponding to each of the three subblocks are higher in caregivers with non-infected PSAC. However, other aspects of infection in PSAC such as intensity of infection, macrohematuria, or microhaematuria were not significantly associated with caregivers’ knowledge. Bathing and washing clothes in the river were also found to be a risk factor for PSAC infection (OR = 2.20, 95%CI: 1.16–4.16 and OR = 2.50, 95%CI: 1.28–4.89, respectively). In addition, not having prior knowledge about schistosomiasis also appeared as another significant risk factor (OR = 2.47, 95%CI: 1.79–3.39).

**Table 7:** Risk factors associated with *S*. *haematobium* infection in PSAC.

Caregiver variable	infected PSAC (N = 68) N (%)	non-infected PSAC (N = 115) N (%)	OR [CI 95%]
Not knowing about schistosomiasis	13 (19.1)	3 (2.6)	2.47 [1.79–3.39]
Not recognising any symptom	32 (47.1)	32 (27.8)	2.17 [1.41–3.32]
Haematuria is not a symptom	50 (73.5)	49 (42.6)	2.36 [1.50–3.71]
Abdominal pain is not a symptom	58 (85.3)	68 (59.1)	2.62 [1.45–4.75]
It cannot cause death	30 (44.1)	37 (32.1)	1.80 [1.16–2.76]
Not knowing source of infection	43 (63.2)	54 (46.9)	3.27 [1.60–6.67]
Believe it cannot be prevented	27 (39.7)	21 (18.8)	2.27 [1.52–3.38]
Avoid river bathing does not prevent it	65 (95.6)	85 (73.9)	4.76 [1.60–14.24]
Believe it cannot be cured	5 (7.3)	3 (2.7)	1.91 [1.10–3.41]
Medicines are not the treatment	22 (32.4)	18 (15.7)	1.70 [1.20–2.47]
Never had schistosomiasis	56 (82.4)	77 (67.0)	1.75 [1.03–2.98]
River bathing	48 (70.6)	60 (52.2)	2.20 [1.16–4.16]
River laundry	52 (76.5)	65 (56.5)	2.50 [1.28–4.89]
Low disease knowledge	66 (97.1)	76 (66.1)	16.93 [3.93–72.82]
Low prevention knowledge	47 (69.1)	46 (40.0)	3.35 [1.77–6.33]
Low treatment knowledge	21 (30.9)	18 (15.7)	2.40 [1.17–4.94]
Low schistosomiasis knowledge	53 (77.9)	37 (32.2)	7.45 [3.72–14.91]

In addition, we found that low schistosomiasis knowledge (OR = 2.24,95%CI: 1.05–4.47), never having heard of schistosomiasis (OR = 2.70, 95%CI: 1.20–6.10), not recognizing the symptoms of schistosomiasis (OR = 2.22, 95%CI: 1.12–4.41), believing you can become infected by eating sugar cane (OR = 2.78, 95%CI: 1.44–5.39), and not knowing how to prevent it (OR = 8.14, 95%CI: 1.14–58.25) were also, among others (Table 8), risk factors for the development of UT morbidity in PSAC.

**Table 8:** Risk factors associated with *S*. *haematobium* morbidity in PSAC.

Caregiver variable	PSAC morbidity (N = 32) N (%)	PSAC w/o morbidity (N = 151) N (%)	OR [CI 95%]
Not knowing about schistosomiasis	5 (15.6)	10 (6.6)	2.7 [1.20–6.10]
Not recognising any symptom	19 (59.4)	41 (27.1)	2.22 [1.12–4.41]
Haematuria is not a symptom	20 (62.5)	66 (43.7)	1.98 [1.02–3.90]
Sugar cane as source of infection	6 (18.7)	15 (9.9)	2.78 [1.44–5.39]
Avoid river bathing does not prevent it	31 (96.9)	94 (62.2)	8.14 [1.14–58.25]
MDA refusal	4 (12.5)	0 (0.0)	1.27 [1.17–1.39]
River bathing	22 (68.7)	67 (44.3)	2.33 [1.04–5.26]
River laundry	27 (84.4)	68 (45.0)	3.9 [1.46–10.63]
Low disease knowledge	30 (93.7)	92 (60.9)	1.16 [1.10–1.25]
Low prevention knowledge	20 (62.5)	62 (41.1)	2.2 [1.02–4.72]
Low schistosomiasis knowledge	19 (59.4)	61 (40.4)	2.24 [1.05–4.77]

## Discussion

To the best of our knowledge, this is the first study on KAP regarding urinary schistosomiasis carried out in Angola.

Knowledge about urogenital schistosomiasis is generally poor, with most respondents not answering half of the survey correctly. Nonetheless, 93.6% had prior knowledge about the existence of schistosomiasis. This high percentage could probably be due to the high prevalence of the disease in that area [15]. While only one person (0.4%) scored high knowledge, 105 (42%) were classified as having low knowledge. Although knowledge in endemic areas varies considerably depending on each of the published studies, low scores are the most common results [19,20,21]. Statistical analyses showed that higher knowledge scores appeared to be correlated with having had a treated schistosomiasis infection in the past (X^2^ = 10.18, *p*-value < 0.01) and a low knowledge score with low socioeconomic status (X^2^ = 29.57, *p*-value < 0.01). Other socioeconomic and demographic characteristics such as literacy, educational level, marital status, or occupation did not show a significant association with the knowledge score. Only two people declared the radio as a source of information on schistosomiasis. Taking into account that 115 households (46.0%) have a radio and that it is a device that does not require electrical installation, so lower-income families can have it at home, the radio is a still untapped tool that could be useful to raise awareness of this disease.

The fact of carrying out the methodology without a self-administrated survey and with open questions allowed us to consider cultural myths that were not planned, such as the thought that eating sugar cane is a source of schistosomiasis infection. Fifty-two people (20.8%) had this belief, which means a double negative impact. On the one hand, people who hold this belief are not aware of the real source of infection, which makes correct prevention practice impossible. On the other hand, efforts to prevent the disease can have an economic impact on sugar cane farmers. A possible explanation is that the cultivation of sugar cane in this area was practiced mainly along the river; in addition, it can be a potential source for other trematodiasis such as human fascioliasis when grown in swampy areas of Africa [22]. In fact, its infection has already been reported in the area [23,24]. However, it is worth highlighting the high knowledge about the schistosomiasis treatment (80%).

Concerning attitude towards schistosomiasis, most respondents scored highest, with 198 people (79.2%) scoring high. In fact, 87.2% of those surveyed would seek for medical care if they detected blood in their urine or experienced abdominal pain, and the average score that the participants gave their doctors in terms of trust was 3.58 out of 5. Nonetheless, this positive attitude is left behind when infected people cannot attend healthcare facilities due to the distance between their residence and health posts and hospitals or due to the cost of the healthcare service (31.4% of cases); in fact, the socioeconomic status was associated with attending a medical consultation (X^2^ = 44.395, *p*-value <0.01). On the other hand, traditional medicine is the only choice for 7.6% of people and 4% expressed that they would not participate in an MDA campaign because they do not trust modern pills, which could result in their children not being able to benefit from MDA campaigns. Unfortunately, MDA campaigns are not being carried out in the province of Benguela and therefore, its community does not have easy access to the drug on a widespread basis at the moment.

Regarding the practices, laundry and bathing in the river appeared to be the most common risky activities, which is consistent with the results published in other studies [6,20,21,25]. 50% of the respondents stated that they urinate in the river, which is a major concern since this makes it difficult to interrupt *S*. *haematobium* transmission as it also happens in other countries [8,21]. With respect to those risky practices, people who come into contact with river water for non-recreational purposes currently have no other option available. In fact, as can be seen in Table 5, approximately 30% of the people who carry out these practices do so only during the dry season, when public water supply systems are interrupted and therefore they have no possibility of access to clean water. Although in most KAP surveys, fishing is one of the highest risk activities [6], this was not the case in our study. The professional activity of cultivating represented twice as many contacts with water as fishing, and this considering that most farmers only cultivate during the rainy season and therefore do not come into contact with the river for their professional activity. This is not surprising if one takes into account that only 6% of the Angolan population work in fishing while 46.7% work in the fields [13]. On the other hand, only 23.2% stated that they carry out prevention activities. Our correlation analysis showed that prevention actions and knowledge of schistosomiasis are significantly associated (X^2^ = 22.59, *p*-value < 0.01).

The results of the analyses to determine the risk factors for *S*. *haematobium* infection among PSAC with respect to their caregivers have revealed that it is linked in many aspects (Table 7). Having bath and laundry in the river appear as common risk factors in other studies on the association between infection of PSAC and their caregivers [11,26,27]. In our study, children under caregivers with poor knowledge of schistosomiasis were more likely to become infected than those with average knowledge, which is consistent with the findings of many other studies [26,27] but surprisingly contrary to the conclusions of one conducted in Malawi [28].

To the best of our knowledge, there are no other studies on the association between UT morbidity due to *S*. *haematobium* in PSAC and their caregivers. Lack of knowledge on how to prevent the disease resulted in the most determining risk factor for the development of UT morbidity in PSAC. Although caregivers’ attitude towards MDA campaigns and river contact was associated with their morbidity, most risk factors were related to their knowledge (Table 8). This evidences that health promotion among caregivers not only protects PSAC from becoming infected, as it is well known [27], but also protects them against developing the disease to a chronic stage. It is worthy highlighting that no significant differences were found in KAP outcomes between caregiver and non-caregiver participants. The results obtained by this study can be used for the design of a health promotion strategy that eliminates the gap between knowledge and practice, providing the environment to achieve a change in behaviour that can have a high impact on the protection of the disease among PSAC.

The main limitations of this study refer to the sampling process. Some parents were reluctant to give their written consent to allow their children to participate in the study on prevalence and morbidity of urogenital schistosomiasis, which explains the relatively small sample size as compared to the population of Cubal. However, our results provide an overview of the general situation of schistosomiasis in the community of Cubal and therefore, the findings and conclusions of the study can contribute to understanding the importance of informing the population about the disease and making municipal and provincial authorities aware of the need to develop and implement programs against schistosomiasis. On the other hand, it has to be noted that the outcomes of our study cannot be generalizable to other regions of the country because some provinces are already conducting disease control programs and MDA campaigns.

## Conclusion

In conclusion, the Cubal population has a medium knowledge about schistosomiasis and a high number of risk practices, and their attitudes towards the prevention and treatment of the disease constitute a promising starting point to improve. In addition, poor knowledge and risk practices of caregivers can represent a risk factor both for the infection of the children in their care and for the development of the disease and its associated morbidity.

Apart from making communities aware to reduce risky practices, viable alternatives should also be offered to improve their living conditions. This is a task that should be carried out mainly by governments, building the necessary infrastructures to provide clean water and sanitation, as well as developing health promotion programs and implementing MDA campaigns. In addition, schools should also provide information on schistosomiasis in order to sensitize SAC to risky practices.

## Consent for publication

Every participant was informed about the type of study and the purpose of its outcomes. Participants (or caregivers in the case of children) signed an informed consent for the inclusion of their children in the study. The informed consent and the survey were provided both in Portuguese and local language (Umbundu).

## References

[1] McManus D. P., Dunne D. W., Sacko M., Utzinger J., Vennervald B. J., Zhou X. N. Schistosomiasis. Nature reviews. Disease Primers, (2018). 4(1), 13. doi: 10.1038/s41572-018-0013-8 30093684

[2] World Health Organization. Ending the neglect to attain the Sustainable Development Goals: a road map for neglected tropical diseases (2020).2021–2030.

[3] World Health Organization. WHO guideline on control and elimination of human schistosomiasis. (2022). 35235279

[4] Colley D. G., Bustinduy A. L., Secor W. E., King C. H. Human schistosomiasis. Lancet, (2014). 383(9936), 2253–2264. doi: 10.1016/S0140-6736(13)61949-2 24698483 PMC4672382

[5] Angles R., Buchon P., Valero M. A., Bargues M. D., Mas-Coma S. One Health Action against Human Fascioliasis in the Bolivian Altiplano: Food, Water, Housing, Behavioural Traditions, Social Aspects, and Livestock Management Linked to Disease Transmission and Infection Sources. International Journal of Environmental Research and Public Health, (2022). 19(3), 1120. doi: 10.3390/ijerph19031120 35162146 PMC8834723

[6] Anyolitho M. K., Poels K., Huyse T., Tumusiime J., Mugabi F., Tolo C. et al. Knowledge, attitudes, and practices regarding schistosomiasis infection and prevention: A mixed-methods study among endemic communities of western Uganda. PLoS neglected tropical diseases, (2022). 16(2), e0010190. doi: 10.1371/journal.pntd.0010190 35196328 PMC8865686

[7] Kayuni S., Lampiao F., Makaula P., Juziwelo L., Lacourse E. J., Reinhard-Rupp J et al. A systematic review with epidemiological update of male genital schistosomiasis (MGS): a call for integrated case management across the health system in sub-Saharan Africa. Parasite epidemiology and control, (2019). 4, e00077. doi: 10.1016/j.parepi.2018.e00077 30662962 PMC6324017

[8] Anyanwu F. C., Ramoteme M., Mabunda J., Henry A., Kwabena K., Nenzhelele F. A quantitative assessment of the level of knowledge, attitude and practices of farmworkers regarding schistosomiasis in a rural community in South Africa. African Journal of Primary Health Care and Family Medicine, (2020). 12(1), 1–8. 10.4102/phcfm.v12i1.2098 PMC7343941 32634007

[9] Dabo A., Badawi H. M., Bary B., Doumbo O. K. Urinary schistosomiasis among preschool-aged children in Sahelian rural communities in Mali. Parasites & Vectors, (2011). 4(1), 1–7. doi: 10.1186/1756-3305-4-21 21338486 PMC3058107

[10] Mazigo H. D., Uisso C., Kazyoba P., Nshala A., Mwingira U. J. Prevalence, infection intensity and geographical distribution of schistosomiasis among pre-school and school aged children in villages surrounding Lake Nyasa, Tanzania. Scientific Reports, (2021). 11(1), 1–11. doi: 10.1038/s41598-020-80317-x 33432079 PMC7801377

[11] Kibira S. P. S., Ssempebwa J. C., Ssenyonga R., Radloff S., Makumbi F. E. Schistosomiasis infection in pre-school aged children in Uganda: a qualitative descriptive study to identify routes of exposure. BMC infectious diseases, (2019). 19(1), 1–10. 30764781 10.1186/s12879-019-3803-z PMC6376787

[12] Instituto Nacional de Estatísticas de Angola Pobreza Multidimensional nos Municípios de Angola. (2019).

[13] República de Angola Avaliação da Estratégia e do Programa do País. (2018).

[14] Mendes E. P., Okhai H., Cristóvão R. E., Almeida M. C., Katondi N., Thompson R., et al. Mapping of schistosomiasis and soil-transmitted helminthiases across 15 provinces of Angola. PLOS Neglected Tropical Diseases, (2022). 16(6), e0010458. doi: 10.1371/journal.pntd.0010458 35771862 PMC9278740

[15] Bocanegra C., Gallego S., Mendioroz J., Moreno M., Sulleiro E., Salvador F., et al Epidemiology of Schistosomiasis and Usefulness of Indirect Diagnostic Tests in School-Age Children in Cubal, Central Angola. PLoS Neglected Tropical Diseases, (2015). 9(10), e0004055. doi: 10.1371/journal.pntd.0004055 26474169 PMC4608768

[16] World Health Organization. *Advocacy*, *communication and social mobilization for TB control*: *a guide to developing knowledge*, *attitude and practice surveys* (No. WHO/HTM/STB/2008.46). World Health Organization. (2008).

[17] UNICEF. MULTIPLE INDICATOR CLUSTER SURVEY MANUAL 2005. (2005).

[18] Principles and recommendations for population and housing censuses (revision 2). New York: United Nations; 2007

[19] Odhiambo G. O., Musuva R. M., Atuncha V. O., Mutete E. T., Odiere M. R., Onyango R. O et al. Low levels of awareness despite high prevalence of schistosomiasis among communities in Nyalenda informal settlement, Kisumu city, western Kenya. PLoS neglected tropical diseases, (2014). 8(4), e2784. doi: 10.1371/journal.pntd.0002784 24699502 PMC3974654

[20] Dawaki S., Al-Mekhlafi H. M., Ithoi I., Ibrahim J., Abdulsalam A. M., Ahmed A et al The Menace of Schistosomiasis in Nigeria: Knowledge, Attitude, and Practices Regarding Schistosomiasis among Rural Communities in Kano State. PloS one, (2015). 10(11), e0143667. doi: 10.1371/journal.pone.0143667 26606264 PMC4659601

[21] Folefac L. N., Nde-Fon P., Verla V. S., Tangye M. N., Njunda A. L., Luma H. N. Knowledge, attitudes and practices regarding urinary schistosomiasis among adults in the Ekombe Bonji Health Area, Cameroon. Pan African Medical Journal, (2018). 29(1), 1–9. 10.11604/pamj.2018.29.161.14980 PMC6057561 30050625

[22] Mas-Coma S., Bargues M. D., Valero M. A. Human fascioliasis infection sources, their diversity, incidence factors, analytical methods and prevention measures. Parasitology (2018). 145 (13, Special Issue), 1665–1699. doi: 10.1017/S0031182018000914 29991363

[23] Mas-Coma S., Valero M. A. Bargues M. D. Human and animal fascioliasis: Origins and worldwide evolving scenario. Clinical Microbiology Reviews, (2022). Ahead of print, 96 pp., 56 figures, 626 references. doi: 10.1128/cmr.00088-19 36468877 PMC9769525

[24] De Alegria MLAR, Colmenares K, Espasa M, Amor A, Lopez I, Nindia A, et al. Prevalence of *Strongyloides stercoralis* and other intestinal parasite infections in school children in a rural area of Angola: a cross-sectional study. American Journal of Tropical Medicine and Hygiene, 2017; 97:1226–1231. 28820707 10.4269/ajtmh.17-0159 PMC5637607

[25] Sady H., Al-Mekhlafi H. M., Atroosh W. M., Al-Delaimy A. K., Nasr N. A., Dawaki S., et al Knowledge, attitude, and practices towards schistosomiasis among rural population in Yemen. Parasites & vectors, (2015). 8(1), 1–13. doi: 10.1186/s13071-015-1050-8 26302747 PMC4548916

[26] Mutsaka-Makuvaza M. J., Matsena-Zingoni Z., Katsidzira A., Tshuma C., Chin’ombe N., Zhou X. N., Webster B et al Urogenital schistosomiasis and risk factors of infection in mothers and preschool children in an endemic district in Zimbabwe. Parasites & vectors, (2019). 12(1), 427. doi: 10.1186/s13071-019-3667-5 31477172 PMC6721289

[27] Sacolo-Gwebu H., Kabuyaya M., Chimbari M. Knowledge, attitudes and practices on schistosomiasis and soil-transmitted helminths among caregivers in Ingwavuma area in uMkhanyakude district, South Africa. BMC infectious diseases, (2019b). 19(1), 1–11. 31438865 10.1186/s12879-019-4253-3 PMC6704662

[28] Moyo V. B., Changadeya W., Chiotha S., Sikawa D. Urinary schistosomiasis among preschool children in Malengachanzi, Nkhotakota District, Malawi: Prevalence and risk factors. Malawi medical journal: the journal of Medical Association of Malawi, (2016). 28(1), 10–14. doi: 10.4314/mmj.v28i1.3 27217911 PMC4864386

